# Synthesis of fluorescent (benzyloxycarbonylamino)(aryl)methylphosphonates

**DOI:** 10.3762/bjoc.10.68

**Published:** 2014-03-28

**Authors:** Michał Górny vel Górniak, Anna Czernicka, Piotr Młynarz, Waldemar Balcerzak, Paweł Kafarski

**Affiliations:** 1 Department of Bioorganic Chemistry, Faculty of Chemistry, Wrocław University of Technology, Wybrzeże Wyspiańskiego 27, 50-370 Wrocław, Poland; 2 Department of Chemistry, University of Opole, pl. Kopernika 11a, 45-040 Opole, Poland; 3 First Department of General, Gastroenterological and Endocrinological Surgery, Wroclaw Medical University, ul. Marii Skłodowskiej-Curie 66, 50-369 Wrocław, Poland

**Keywords:** aminophosphonates, Oleksyszyn reaction, organophosphorus, Z-aminophosphonate esters

## Abstract

The synthesis of a library of structurally variable aromatic esters of (benzyloxycarbonylamino)(aryl)methylphosphonic acids is described by means of the Oleksyszyn reaction. The library was enlarged by the application of a Suzuki–Miayra approach and by preparation of mixed esters.

## Introduction

Screening of the activity of large libraries of fluorogenic substrates of chosen enzymes is an emerging approach to determine their substrate preferences and thus to provide a set of data useful for the preparation of their selective inhibitors [1–5]. Such fluorogenic probes have been also used for profiling the proteolytic secretomes with the obtained profiles being useful as diagnostic tools [6–8]. Also conjugates of drugs with fluorescent probes have been used as so called theranostics (combination of therapeutics and diagnostics) [9–10].

Diaryl esters of α-aminoalkanephosphonic acids and their short peptides are a class of well-established inhibitors of serine proteases [11–12]. Their mechanism of action involves phosphonylation of the active-site of these enzymes with simultaneous release of the appropriate phenol [13–14]. Therefore, the synthesis of such inhibitors carrying fluorescent probes in their side chains or in the ester phosphonate moieties might find an application in constructing fluorescent probes for studying structural requirements of enzymes having serine in their active sites (proteinases and phosphatases) or to study their elevated level in various tissues. Thus, their libraries may serve for the construction of diagnostic tools. A similar approach has been recently patented as a mean to differentiate lipases and esterases [15].

The objective of this paper is to evaluate the utility of a popular three component reaction of triaryl phosphites with aldehydes and benzyl carbamates (the so called Oleksyszyn reaction) for the synthesis of fluorescent (benzyloxycarbonylamino)(aryl)methylphosphonates.

## Results and Discussion

### Synthesis of diaryl (benzyloxycarbonylamino)(phenyl)methylphosphonates

**Scheme 1:**
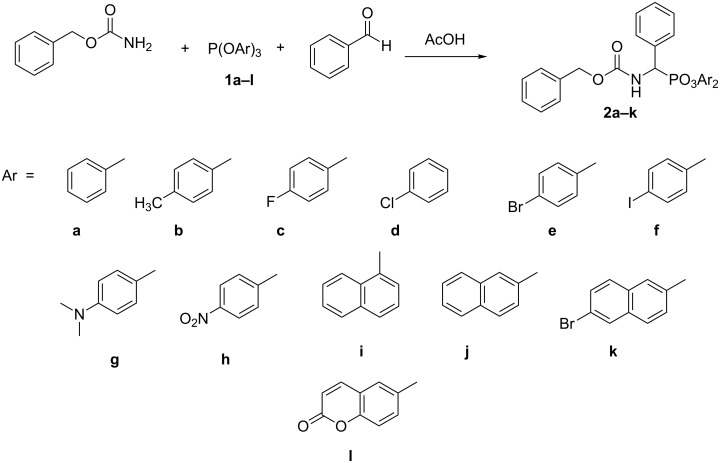
Diaryl (benzyloxycarbonylamino)(phenyl)methylphosphonates.

Diaryl esters of Z-protected aminobenzylphosphonic acid were obtained by using the classical three-component amidoalkylation procedure described by Oleksyszyn et al. (Scheme 1) [14].

First, appropriate triaryl phosphites have to be synthesized. They were obtained by refluxing stoichiometric quantities of phosphorus trichloride with the appropriate phenol (molar ratio 1:3) in acetonitrile [16–17]. The desired phosphites deposited from acetonitrile as solids or oils and did not require further purification. Corresponding reactions carried out in different solvents (toluene, benzene or diethyl ether in the presence of butyllithium in hexane) or with phosphorus tribromides gave far less satisfactory results because the obtained crude products were difficult to purify.

The obtained phosphites were reacted with benzaldehyde and benzyl carbamate, according to literature [18–20], providing the desired diaryl (benzyloxycarbonylamino)(phenyl)methylphosphonates (Scheme 1) with good yields (59–86%). Unfortunately, the reaction of phosphite obtained from 7-hydroxycoumarine failed, since it appeared to be unstable upon harsh reaction conditions and underwent decomposition as seen by ^31^P NMR. Therefore, we have decided to apply a more delicate procedure described recently by Goldeman and Soroka [21]. According to this procedure the reaction was carried out in dry chloroform in the presence of catalytic amounts of tetrafluoroboric acid. Unfortunately, tri(7-hydroxycoumaric) phosphite also underwent decomposition under these conditions.

The diaryl esters **2** exhibit restricted rotation around the C–N bound of their carbamate group. As a consequence, they exist in solution in *cis*- and *trans-*forms, with the equilibrium strongly shifted towards the formation of *trans*-isomers [22].

### Synthesis of diaryl (benzyloxycarbonylamino)(aryl)methylphosphonates

**Scheme 2:**
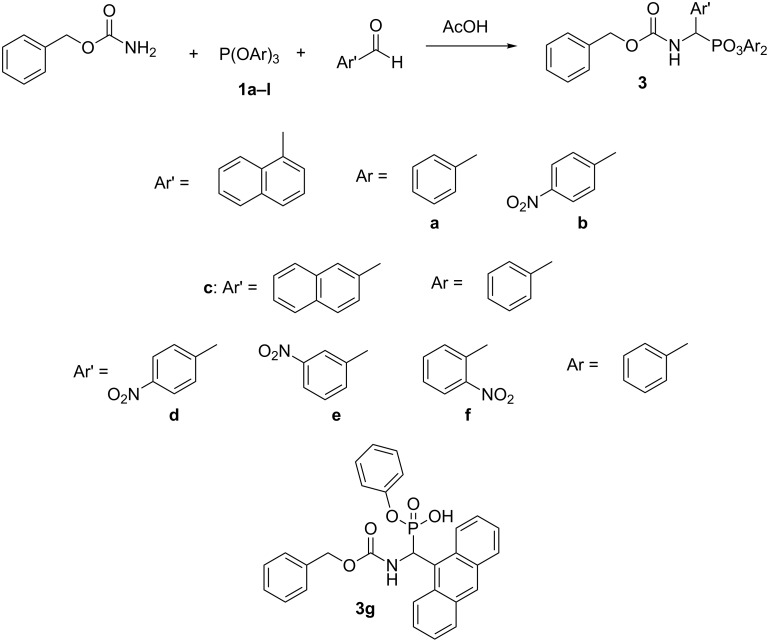
Diaryl (benzyloxycarbonylamino)(aryl)methylphosphonates.

By applying the same procedure we have synthesized a small library of diaryl esters of aromatic Z-aminophosphonates (Scheme 2). They were obtained in satisfactory yields (55–84%). When using 5-anthracenylaldehyde a complex mixture of products was obtained and the product was isolated by column chromatography in a low yield of 1% as the monophenyl ester.

**Scheme 3:**
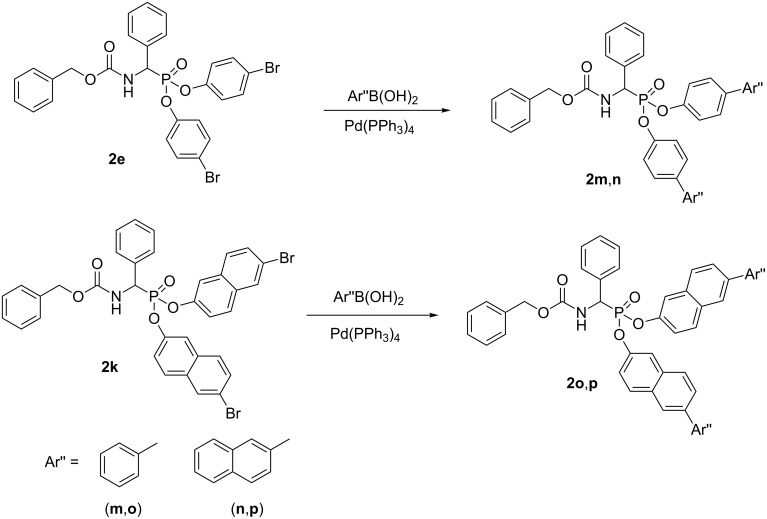
Diaryl (benzyloxycarbonylamino)(aryl)methylphosphonates obtained by Miyaura–Suzuki approach.

This library was enlarged by application of the Suzuki–Miyaura approach with compounds **2e** and **2k** being chosen as substrates (Scheme 3) [23–24]. Despite the enormous number of data considering application of this reaction, according to the best of our knowledge, there is no report on its application to synthesize phosphorus esters. After dissolving the substrates the catalyst was added to the reaction mixture and it was carried under reflux for 6 h. Optimization of the reaction conditions revealed that a mixture of dioxane and water (out of: dioxane, acetonitrile, chloroform and acetonitrile/water mixture) appeared to be the best solvent with 5% of Pd(PPh_3_)_4_ serving as optimal catalyst.

### Synthesis of mixed esters of (benzyloxycarbonylamino)(aryl)methylphosphonates

**Scheme 4:**
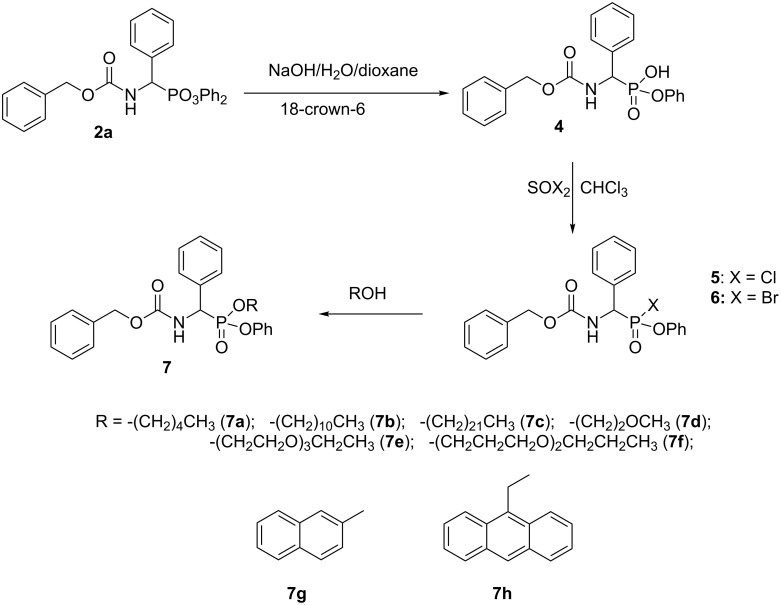
Synthesis of mixed esters.

Also a small library of mixed esters was obtained. Using the procedures worked out in our laboratory diphenyl (benzyloxycarbonylamino)(phenyl)methylphosphonate (**2a**) was converted into the monoester by hydrolysis with aqueous potassium hydroxide in the presence of 18-crown-6 [25–26]. The obtained monoester **4** was transformed into chloride **5** or bromide **6**, which were used in the next step of the synthesis without purification (Scheme 4). Upon halogen introduction a new chirality center is formed at the phosphorus atom and the products **5** and **6** were obtained as unequimolar mixtures of diastereoisomers (45:55 and 42:58 respectively). Upon reaction with an excess of aliphatic alcohol mixed esters of reversed configuration were obtained, as indicated by the reversed order of the ^31^P NMR peaks. This is in agreement with the mechanism of this reaction, which proceeds with inversion of the configuration at the phosphorus atom. Upon purification of the obtained compounds **7**, either by crystallization or column chromatography, a significant enrichment in one of the diastereomers had been observed. For example, compound **7b** obtained as a 55:45 molar mixture of stereoisomers after purification by means of chromatography was obtained as 85:15 molar mixture.

The approach to obtain mixed esters bearing two aromatic moieties is far more difficult as shown by reaction of chloride **5** with 2-naphthol providing compound **7g**. This reaction was carried out in chloroform in the presence of triethylamine.

### Fluorescence

Fluorescence of the representative examples of the obtained compounds was measured upon irradiation of two wavelengths of 254 and 366 nm. Compounds **2n** and **3g** exhibited strong fluorescence under both conditions. The remaining phosphonates either show a weak (**2h**, **3b**, **3d** and **3e**) fluorescence when irradiated with 254 nm or exhibited the lack of fluorescence (**2f**, **2k**, **2m**, **2o**, **3a**, **3c**, **4**, **7a**, **7d**, **7e**, **7g**).

## Supporting Information

File 1 Experimental procedures and analytical data and NMR spectra.
