# Supplementary material for: Synthesis of fluorescent (benzyloxycarbonylamino)(aryl)methylphosphonates
Source: Beilstein J Org Chem. 2014 Mar 28;10:741–5. doi: 10.3762/bjoc.10.68 (PMC3999870; doi:10.3762/bjoc.10.68)
Supplement: File 1 — Experimental procedures and analytical data and NMR spectra. [file Beilstein_J_Org_Chem-10-741-s001.pdf]

Supporting Information

for

Synthesis of fluorescent (benzyloxycarbonylamino)(aryl)methylphosphonates

Michał Górny vel Górniak[1,2] , Anna Czernicka[1] , Piotr Młynarz[1] , Waldemar Balcerzak[3] and Paweł Kafarski [1,2,*]

Address: [1]Department of Bioorganic Chemistry, Faculty of Chemistry, Wrocław University of Technology, Wybrzeże Wyspiańskiego 27, 50-370 Wrocław, Poland, [2]Departmnent of Chemistry, University of Opole, pl. Kopernika 11a, 45-040 Opole, Poland and [3]First Department of General, Gastroenterological and Endocrinological Surgery, Wroclaw Medical University, ul. Marii Skłodowskiej-Curie 66, 50-369 Wrocław, Poland

Email: Paweł Kafarski* - pawel.kafarski@pwr.wroc.pl

* Corresponding author

Experimental procedures and analytical data and NMR spectra.

Table of contents:

- General information S2
- Experimental procedures with copies of representative NMR spectra S2
- Fluorescence studies S19
- References S20

General information

All solvents and reagents were purchased from commercial suppliers (Aldrich, Sigma, Merck, POCh), were of analytical grade and were used without further purification. Unless otherwise specified the solvents were removed with a rotary evaporator. Infrared spectra were measured on a 1600 FT-IR Perkin-Elmer spectrometer. NMR experiments were performed on Bruker DRX AVANCE^{TM} 300 MHz and Bruker AVANCE^{TM} 600 MHz sperctrometers. Measurements were made in $CDCl_3$ (99.5 at. % D) or D_2O (99.8 at. % D) solutions at temperature 300 K, all solvents were supplied by Dr Glaser AG (Basel, Switzerland). Chemical shifts are reported in parts per million relative to TMS or 85% H_3PO_4 used as external standards, and coupling constants are reported in Hertz. Melting points were determined on an Electrothermal 9200 apparatus and are reported uncorrected. Elemental analyses were performed at the Chemistry Department of the University of Wroclaw on a Perkin Elmer 2400 CHN analyser. Electrospray mass spectra were recorded at the Chemistry Department of the University of Wroclaw using a Finnigan Mat TSQ 700 Electrospray mass spectrometer.

Experimental procedures with copies of representative NMR spectra

Triaryl phosphites 1

To the solution of apprioprate phenol (50 mmol) in dry acetonitrile (200 mL) phosphorus trichloride (1.47 mL, 16 mmol) was added dropwise and the obtained mixture was refluxed for 5 h. After cooling the product precipitated or deposited as an oil. It was washed with acetonitrile and dried in dessicator. Phosphites were characterized by their ^{31}P NMR spectra (presence of only a single phosphorus signal) and used directly after synthesis.

Tris(4-methylphenyl) phosphite (**1b**)*;* 58% yield; yellow oil, ^{31}PNMR ($CDCl_3$): δ = 129.2 ppm [1].

Tris(4-fluorophenyl) phosphite (**1c**)*;* 58% yield; dense colorless oil, ^{31}PNMR ($CDCl_3$): δ = 128.2 ppm.

Tris(4-chlorophenyl) phosphite (**1d**)*;* 88% yield; yellowish oil, ^{31}PNMR ($CDCl_3$): δ = 127.6 ppm [1].

Tris(4-bromophenyl) phosphite (**1e**)*;* 89% yield; yellowish oil, ^{31}PNMR ($CDCl_3$): δ = 125.2 ppm.

Tris(4-iodophenyl) phosphite (**1f**)*;* 78% yield; yellowish oil, ^{31}PNMR ($CDCl_3$): δ = 126.6 ppm.

Tris(4-N,N-dimethylaminophenyl) phosphite (**1g**)*;* 89% yield; brownish oil, ^{31}PNMR ($CDCl_3$): δ = 127.6 ppm.

Tris(4-nitrophenyl) phosphite (**1h**);95% yield; yellow crystals mp. 175-176 °C (lit. mp. 174-176 °C), ^{31}PNMR ($CDCl_3$): δ = 126.3 ppm [2].

Tris(1-naphthyl) phosphite (**1i**)*;* 60% yield; white crystals mp. 201-203 °C (lit. m.p. not given [3]), ^{31}PNMR ($CDCl_3$): δ = 131.1 ppm.

Tris(2-naphthyl) phosphite (**1j**); 89% yield; yellow crystals mp. 199-201 °C, ^{31}PNMR ($CDCl_3$): δ = 129.4 ppm.

Tris(6-bromo-2-naphthyl) phosphite (**1k**)*;* 57% yield; yellow oil, ^{31}P NMR ($CDCl_3$): δ = 128.6 ppm.

Tris(7-hydroxycumaryl phosphite (**1l**); 78% yield; yellow crystals mp. 187-190 °C, ^{31}PNMR ($CDCl_3$): δ = 127.1 ppm.

***Diaryl (benzyloxycarbonylamino)(phenyl)methylphosphonates* 2a–k**

Benzyl carbamate (4.53 g, 30 mmol), triarylphosphite (30 mmol) and benzaldehyde (4.53 mL, 45 mmol) were dissolved in acetic acid (100 mL) and the obtained mixture was

refluxed for 2 h. Then the acetic acid was evaporated and the oily residue was dissolved in a small volume of acetone (10–20 mL depending on product), several drops of hexane were added and left for crystallization at 4 °C.

Diphenyl (benzyloxycarbonylamino)(phenyl)methylphosphonate (**2a**); 86% yield; white solid mp. 161-163^{0}C (lit mp. 138-140^{0}C [4]); 85:15 *trans*/*cis* ratio; ^{31}PNMR ($CDCl_3$): δ= 15.52 & 15.46 ppm.; ^{1}HNMR ($CDCl_3$): δ= 5.04 & 5.13 (2H, d, *J* 15 Hz, C<u>H</u>$_2$Ph), 5.56 (1H, d, J_{PH} 22 Hz, *J* 9 Hz, C<u>H</u>P), 5.84 (1H, bd, *J* 9 Hz, N<u>H</u>), 6.80 (2H, d, *J* 9 Hz, Ar), 7.05-7.47 ppm (18H, m, Ar)

Bis(p-methylphenyl) (benzyloxycarbonylamino)(phenyl)methylphosphonate (**2b**); 74% yield; white solid mp. 216-219^{0}C; 86:14 *trans*/*cis* ratio; ^{31}PNMR ($CDCl_3$): δ= 14.56 & 14.43 ppm.; ^{1}HNMR ($CDCl_3$): δ= 3.38 & 3.55 (6H, PhC<u>H</u>$_3$), 5.10 & 5.13 (2H, d, J_{AB} 11 Hz, C<u>H</u>$_2$Ph), 5.60 (1H, dd, J_{PH} 24 Hz, J 9 Hz, C<u>H</u>P), 5.89 (1H, bd, J 9 Hz, N<u>H</u>), 6.87 (2H, d, *J* 9 Hz, Ar), 7.12-7.69 ppm (16H, m, Ar) ppm.

Bis(p-fluorophenyl) (benzyloxycarbonylamino)(phenyl)methylphosphonate (**2c**); 50% yield; white solid mp. 189-193^{0}C; 93:7 *trans*/*cis* ratio; ^{31}PNMR ($CDCl_3$): δ= 15.72 & 15.64 ppm.; ^{1}HNMR ($CDCl_3$): δ= 5.06 (2H, d, *J* 15 Hz, C<u>H</u>$_2$Ph), 5.71 (1H, d, J_{PH} 22 Hz, *J* 9 Hz, C<u>H</u>P), 5.96 (1H, bd, *J* 9 Hz, N<u>H</u>), 7.25-7.82 ppm (18 H, m, Ar) ppm. HRMS (DMSO, TOF MS ESI$^+$): MH$^+$, found 532.1322 [MNa$^+$], $C_{27}H_{22}F_2NO_5PNa$ requires 532.4301

Bis(p-chlorophenyl) (benzyloxycarbonylamino)(phenyl)methylphosphonate (**2d**); 62% yield; white solid mp. 169-171^{0}C; 93:7 *trans*/*cis* ratio; ^{31}PNMR ($CDCl_3$): δ= 14.87 & 14.69 ppm.; ^{1}HNMR ($CDCl_3$): δ= 5.05 & 5.16 (2H, d, J_{AB} 12 Hz, C<u>H</u>$_2$Ph), 5.66 (1H, dd, J_{PH} 22 Hz, *J* 10 Hz, C<u>H</u>P), 5.77 (1H, bd, *J* 10 Hz, N<u>H</u>), 6.78 & 6.88 (4H, d, *J* 8 Hz, Ar), 7.25-7.65 ppm

(14H, m, Ar) ppm; HRMS (DMSO, TOF MS ESI$^+$): found 565.92 [MNa$^+$], $C_{27}H_{22}Cl_2NO_5PNa$ requires 565.3397.

Bis(p-bromophenyl) (benzyloxycarbonylamino)(phenyl)methylphosphonate (**2e**); 72% yield; white solid mp. 157-162^{0}C; 92:8 *trans*/*cis* ratio; ^{31}PNMR ($CDCl_3$): δ= 16.01 & 15.89 ppm.; ^{1}HNMR ($CDCl_3$): δ= 5.06 & 5.18 (2H, d, J_{AB} 13 Hz, C<u>H</u>$_2$Ph), 5.59 (1H, d, J_{PH} 22 Hz, *J* 9 Hz, C<u>H</u>P), 5.83 (1H, bd, J 9 Hz, N<u>H</u>), 6.99 (2H, d, J 8 Hz, Ar), 7.28-7.48 ppm (16H, m, Ar) ppm. HRMS (DMSO, TOF MS ESI$^+$): found 654.0002 [MNa$^+$], $C_{27}H_{22}Br_2NO_5PNa$ requires 654.2415.

Bis(p-iodophenyl) (benzyloxycarbonylamino)(phenyl)methylphosphonate (**2f**)

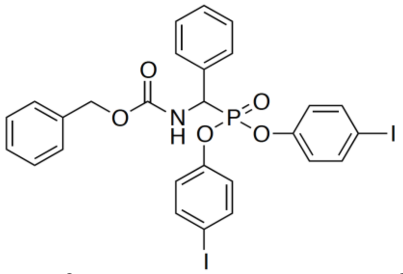

74% yield; white solid mp. 162-165 ^{0}C; 92:8 *trans*/*cis* ratio; ^{31}PNMR (d_6DMSO): δ= 14.95 & 14.70 ppm.; ^{1}HNMR ($CDCl_3$): δ= 5.05 & 5.13 (2H, d, J_{AB} 12 Hz, C<u>H</u>$_2$Ph), 5.63 (1H, dd, J_{PH} 22 Hz, *J* 8 Hz, C<u>H</u>P), 6.92 (2H, d, J 8 Hz, Ar), 7.29-7.69, (16H, m, Ar), 8.91 (1H, d, *J* 8 Hz, NH); ^{13}C NMR ($CDCl_3$): δ= 66.77, 53.31 (d, *J 1*55.5 Hz), 123.09, 123.12, 123.19, 128.52, 128.87, 128.89, 134.40, 137.02, 139.02, 139.09, 150.17, 136.38 ppm. HRMS (DMSO, TOF MS ESI$^+$): found 747.9223 [MNa$^+$], $C_{27}H_{22}I_2NO_5PNa$ requires 748.2422.

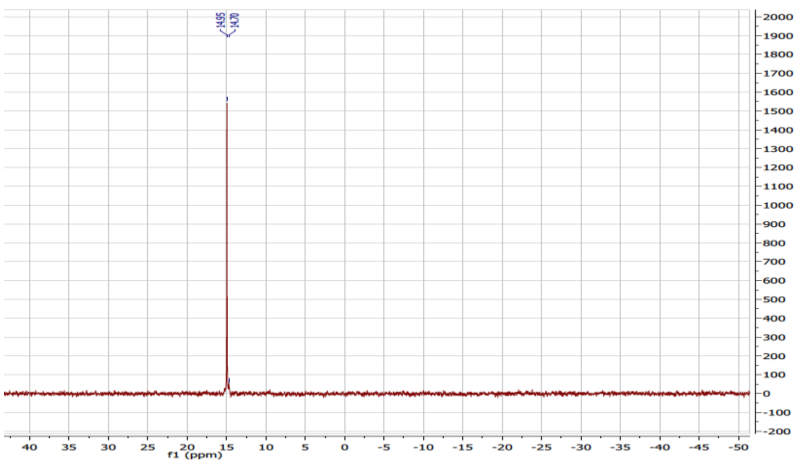

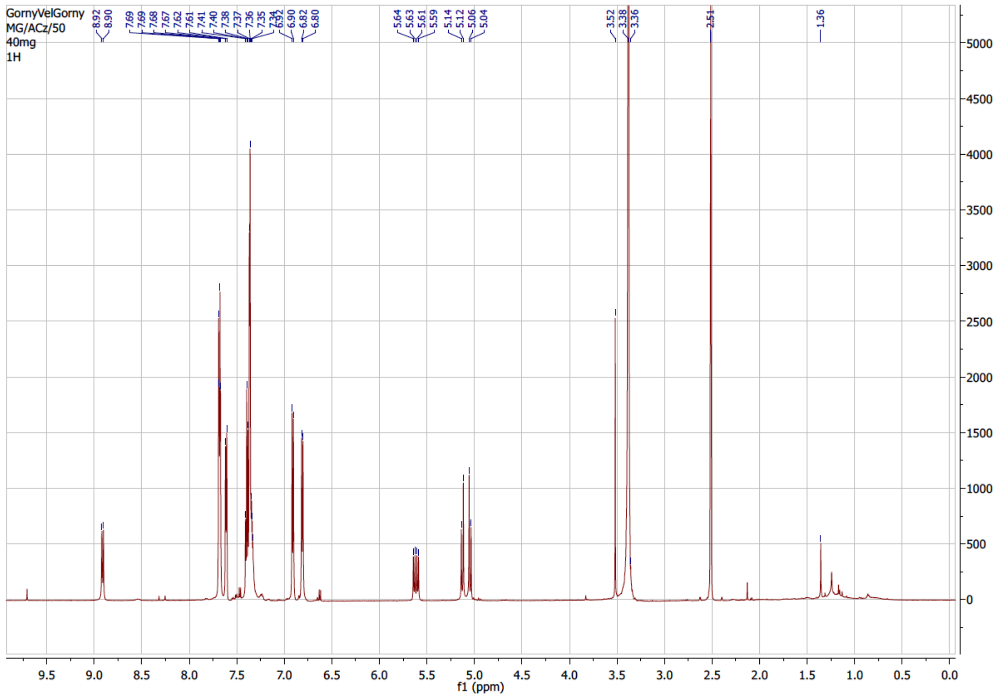

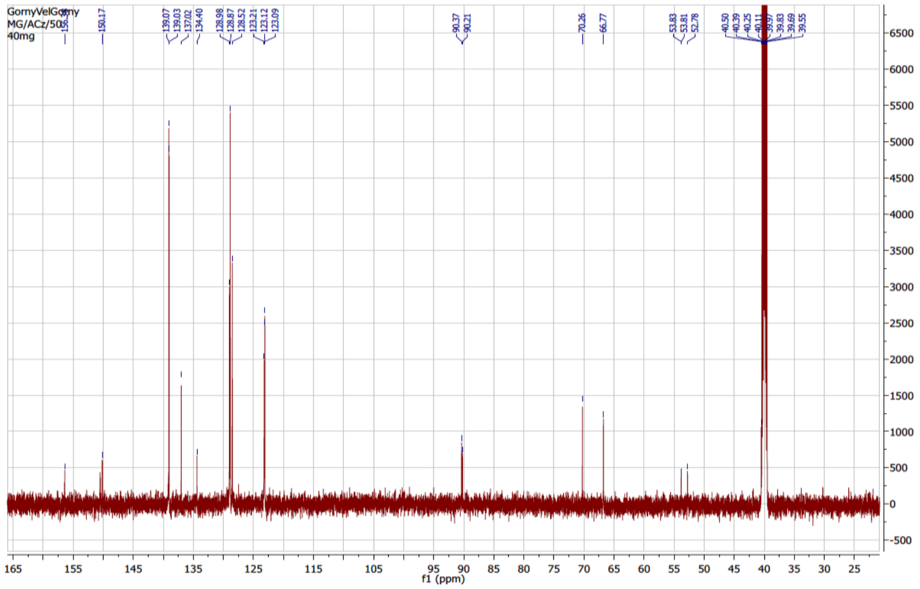

Bis(N,N-dimethylaminophenyl) (benzyloxycarbonylamino)(phenyl)methylphosphonate (**2g**); 59% yield; white solid mp. 168-171^{0}C; 89:11 *trans/cis* ratio; ^{31}PNMR ($CDCl_3$): δ= 14.12 & 13.93 ppm.; ^{1}HNMR ($CDCl_3$): δ= 3.21-3.52 (12H, m), 5.07 & 5.13 (2H, d, J_{AB} 12 Hz, C<u>H</u>$_2$Ph), 5.76 (1H, dd, J_{PH} 24 Hz, J 9 Hz, C<u>H</u>P), 5.83 (1H, bd, *J* 9 Hz, N<u>H</u>), 6.79 (2H, d, J 9 Hz, Ar), 7.31-7.88 ppm (16H, m, Ar) ppm.

Bis(p-nitrophenyl) (benzyloxycarbonylamino)(phenyl)methylphosphonate (**2h**)*;* 68% yield; white solid mp. 175-176^{0}C; 98:2 *trans*/*cis* ratio; ^{31}PNMR ($CDCl_3$): δ= 14.54 & 14.45 ppm.; ^{1}HNMR ($CDCl_3$): δ= 5.19 & 5.25 (2H, d, J_{AB} 12 Hz, CH_2Ph), 5.69 (1H, dd, J_{PH} 22 Hz, *J* 10 Hz, CHP), 5.88 (1H, d, *J* 10 Hz, NH), 6.99 (2H, d, *J* 9 Hz, Ar), 7.24-7.48 (12H, m, Ar), 8.09 & 8.16 ppm (4H, m, *J* 9 Hz) ppm. HRMS (DMSO, TOF MS ESI^{+}): found 586.4401 [MNa^{+}], $C_{27}H_{22}N_3O_9PNa$ requires 586.4446

Bis(1-naphthyl) (benzyloxycarbonylamino)(phenyl)methylphosphonate (**2i**)*;* 49% yield; white solid mp. 186-188^{0}C; 95:5 *trans*/*cis* ratio; ^{31}PNMR ($CDCl_3$): δ= 15.21 & 15.08 ppm.; ^{1}HNMR (d_6DMSO): δ= 5.04 & 5.08 (2H, d, *J* 12 Hz, CH_2Ph), 5.72 (1H, d, J_{PH} 22 Hz, *J* 10 Hz, CHP), 6.01 & 6.04 (1H, bd, *J* 10 Hz, NH), 6.98-7.01 ppm (24H, m, Ar) ppm. HRMS (DMSO, TOF MS ESI^{+}): found 598.0582 [MNa^{+}], $C_{35}H_{30}NO_5PNa$ requires 598.5831.

Bis(2-naphthyl) (benzyloxycarbonylamino)(phenyl)methylphosphonate (**2j**); 72% yield; white solid mp. 172-173^{0}C; 93:7 *trans*/*cis* ratio; ^{31}PNMR ($CDCl_3$): δ= 14.82 & 14.66 ppm.; ^{1}HNMR ($CDCl_3$): δ= 5.05 & 5.10 (2H, d, J_{AB} 12 Hz, CH_2Ph), 5.76 (1H, dd, J_{PH} 24 Hz, *J* 9 Hz, CHP), 5.96 (1H, bd, *J* 9Hz, NH), 6.99-7.82 ppm (24H, m, Ar) ppm.

Bis(6-bromo-2-naphthyl) (benzyloxycarbonylamino)(phenyl)methylphosphonate (**2k**)

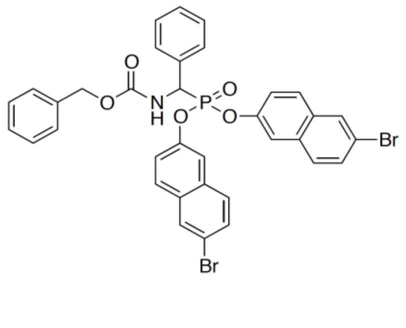

66% yield; white solid mp. 186-189^{0}C; 95:5 *trans*/*cis* ratio; ^{31}PNMR (d_6DMSO): δ= 15.21 & 15.03 ppm.; ^{1}HNMR ($CDCl_3$): δ= 5.04 & 5.09 (2H, d, J_{AB} 12 Hz, $C\underline{H}_2Ph$), 5.63 (1H, d, J_{PH} 22 Hz, *J* 9 Hz, $C\underline{H}P$), 5.96 (1H, bd, *J* 9Hz, $N\underline{H}$), 6.89-7.86 ppm (22H, m, Ar) ppm; HRMS (DMSO, TOF MS ESI$^+$): found 758.0043 [MNa$^+$], $C_{35}H_{30}Br_2NO_5PNa$ requires 758.3913.

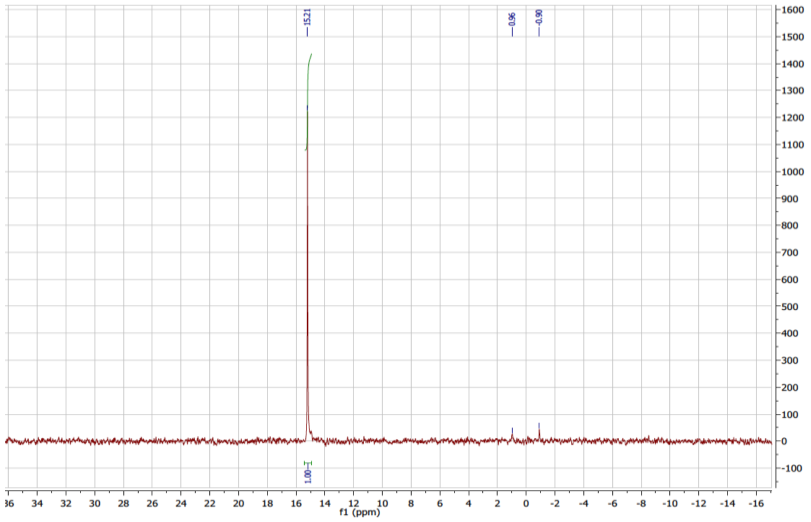

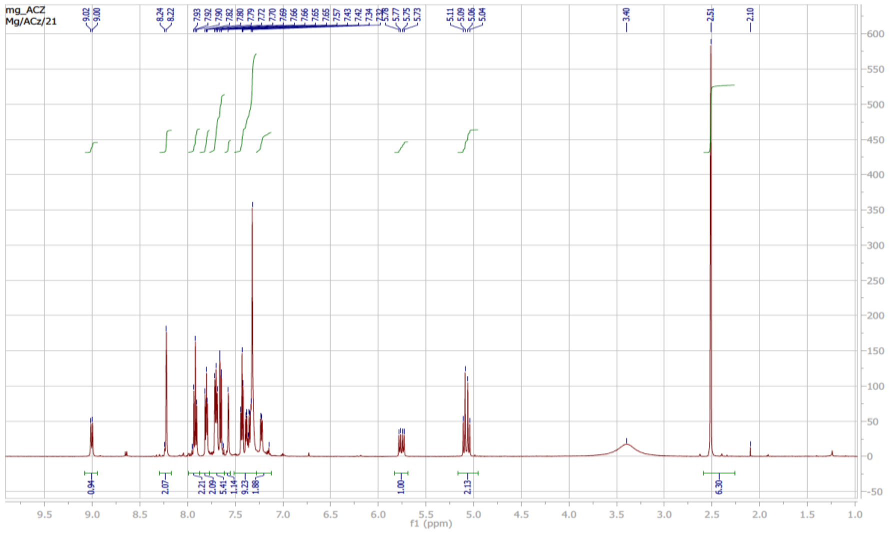

***(Benzyloxycarbonylamino)(aryl)methylphosphonates* 3**

Benzyl carbamate (2.0 g, 13.2 mmol), triaryl phosphite (13.2 mmol) and appropriate aldehyde (1.98 mmol) were dissolved in acetic acid (45 mL) and the obtained mixture was refluxed for 2 h and left overnight. Then acetic acid was evaporated and the oily residue

dissolved in small volume of acetone (5–20 mL depending on product), several drops of hexane were added and left for crystallization at 4 °C.

Diphenyl (benzyloxycarbonylamino)(1-naphthyl)methylphosphonate (**3a**)

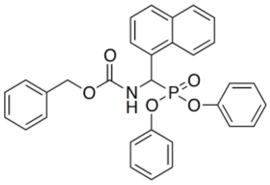

55% yield, mp.178-180^{0}C; ^{31}PNMR ($CDCl_3$): δ= 15.27 ppm.; ^{1}HNMR (d_6DMSO): δ= 5.01 & 5.09 (2H, d, J_{AB} 12.1 Hz, C<u>H</u>$_2$Ph), 6.099 (1H, bm, C<u>H</u>P), 7.15- 8.05 (m, 7H), 8,51 (1H, bm, N<u>H</u>) ppm. HRMS (DMSO, TOF MS ESI$^+$): found 546.1523 [MNa$^+$], $C_{31}H_{26}NO_5PNa$ requires 546.5082.

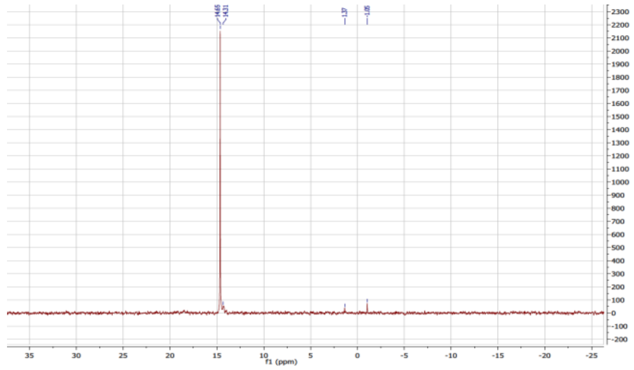

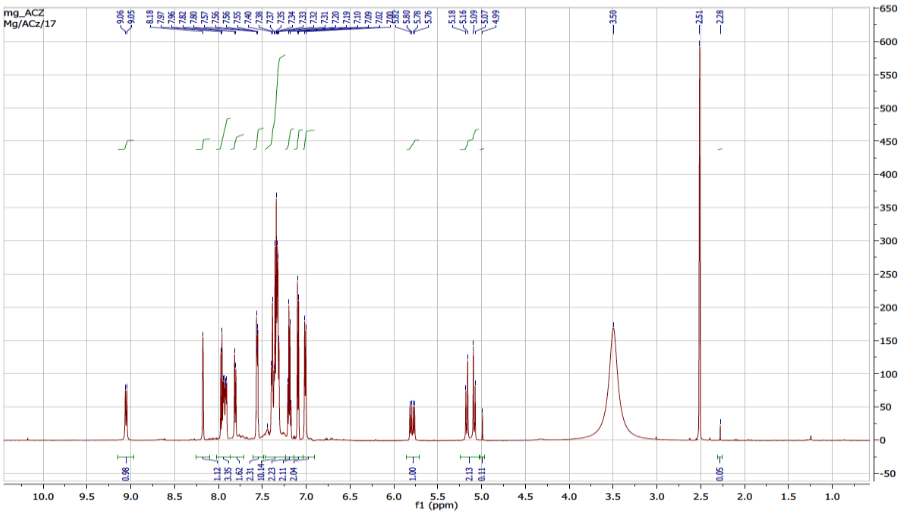

Bis(p-nitrophenyl) (benzyloxycarbonylamino)(2-naphthyl)methylphosphonate (**3b**); 48% yield; mp.168-171^{0}C; ^{31}PNMR ($CDCl_3$): δ= 14.91 ppm.; ^{1}HNMR (d_6DMSO): δ= 5.01§ & 5.09 (2H, d, J_{AB} 11 Hz, C<u>H</u>$_2$Ph), 6.06 (1H, bd, *J* 9.3 Hz, N<u>H</u>), 6.58 (1H, dd, J_{PH} 21.0 Hz, *J* 9.3 Hz, C<u>H</u>P), 7.15- 8.05 (m, 7H), 8,51 (1H, bm, N<u>H</u>) ppm.

Diphenyl (benzyloxycarbonylamino)(2-naphthyl)methylphosphonate (**3c**); 68% yield; 186-189^{0}C; 98:2 trans/cis ratio; ^{31}PNMR ($CDCl_3$): δ= 14.40 & 14.31 ppm.; ^{1}HNMR ($CDCl_3$): δ= 5.08 & 5.17 (2H, d, J_{AB} 12 Hz, C<u>H</u>$_2$Ph), 5.69 (1H, dd, J_{PH} 22 Hz, *J* 9 Hz, C<u>H</u>P), 7.00-7.97 ppm (22H, m, Ar), 9.06 (1H, bd, J 9Hz, N<u>H</u>) ppm. HRMS (DMSO, TOF MS ESI$^+$): found 546.1454 [MNa$^+$], $C_{31}H_{26}NO_5PNa$ requires 546.5082.

Diphenyl (benzyloxycarbonylamino)(p-nitrophenyl)methylphosphonate (**3d**); 84% yield; mp. 164-165^{0}C; ^{31}PNMR ($CDCl_3$): δ= 12.82 ppm.; ^{1}HNMR ($CDCl_3$): δ= 5.12 (2H, dd, *J* 12.0 Hz, C<u>H</u>$_2$Ph); 5.69 (1H, dd, J_{PH} 23.6 Hz, *J* 9.5 Hz, C<u>H</u>P); 6,49 (1H, bd, *J* 15Hz, N<u>H</u>), 6.92 (2H d, *J* 9 Hz, Ar), 6.92 (2H d, *J* 7.8 Hz, Ar), 7.12-7.34 (11H, m, Ar), 7.68 & 8.19 (2H each, d, *J* 8.4Hz, nitroaryl) ppm.

Diphenyl (benzyloxycarbonylamino)(m-nitrophenyl)methylphosphonate (**3e**); 76% yield; mp. 135-137^{0}C; ^{31}PNMR ($CDCl_3$): δ= 12.76 ppm.; ^{1}HNMR ($CDCl_3$): δ= 5.10 & 5.16 (2H, d, J_{AB} 12.2 Hz, C<u>H</u>$_2$Ph), 5.68 (1H, dd, J_{PH} 23.2 Hz, *J* 9.1 Hz, C<u>H</u>P), 6.28 (1H, bd, *J* 9.1 Hz, N<u>H</u>), 6.93-7.36 (15H, m, Ar); 7.52 & 7.84 (1H each, t, *J* 7.2 Hz, nitroaryl), 8.17 (d, 1H, *J* 7.2 Hz, nitroaryl), 8.36 ppm (1H, s, nitroaryl) ppm.

Diphenyl (benzyloxycarbonylamino)(o-nitrophenyl)methylphosphonate (**3f**); 73% yield; mp. 141-143^{0}C; ^{31}PNMR ($CDCl_3$): δ= 12.88 ppm.; ^{1}HNMR ($CDCl_3$): δ= 5.08 & 5.17 (2H, dd, J_{AB} 12.1 Hz, C<u>H</u>$_2$Ph), 6,32, 1H (bd, *J* 9.0 Hz, N<u>H</u>), 6.74-6.90 (3H, m, C<u>H</u>P overlapped with

aryls), 7.10-7.34 (13H, m, Ar), 7.47 &7.60 (t, 1H each, *J* 7.1 Hz, t, nitoaryl), 7.74 & 8.08 (d, 1H each, d, *J* 7.1 Hz, nitoraryl) ppm.

Phenyl ((benzyloxycarbonylamino)anthracen-9-yl)methylphosphonate (**3g**); 1% yield; dcomp. At 250^{0}C; ^{31}PNMR ($CDCl_3$): δ= 14.43 ppm.; ^{1}HNMR (d_6DMSO): δ= 4.99 (1H, d, J_{PH} 27.9 Hz, C<u>H</u>P), 5.16 & 5.19 (2H, d, J_{AB} 21 Hz, C$\underline{H}_2$Ph); 6.72 (1H, d, *J* 7.8 Hz, Ar); 6.82 (1H, d, *J* 7.8 Hz, Ar); 6.94-7.01(1H m, Ar); 7.03-7.12 (3H, m, Ar); 7.17 (2H, t, *J* 7.8 Hz, Ar); 7.32 (1H, t, J=7.6 Hz, Ar); 7.36-7.49 (6H, m, Ar); 7.55 (2H, d, *J* 7.4 Hz, Ar); 7.56 (1H, d, *J* 7.7 Hz, Ar); 7.69 ppm (1H, d, *J* 6.9, Ar) ppm.

Diaryl (benzyloxycarbonylamino)(aryl)methylphosphonates (**2m–p**) ***prepared by Miyaura–Suzuki reaction***

Phosphonate ester **2e** or **2k** (5 mmol) was dissolved in dioxane/water mixture (3:1, 25 mL) and phenylboronic (1.22 g, 10 mmol) or naphthylboronic acid (1.72 g, 10 mmol) was added, followed by addition of $K_3PO_4{\cdot}H_2O$ (0.2 g). Then tetrakis(triphenylphosphine)palladium(0) (4–5 mol %) was added and the mixture refluxed for 5 h. After cooling to room temperature catalyst was removed by filtration and dioxane emoved on rotary evaporator. To the residue water was added (20 mL) and the product was extracted three times with chloroform (10 mL portions). In order to avoid formation of emulsion an additional portion of $K_3PO_4{\cdot}H_2O$ (2.0 g) was dissolved in the aqueous solution. Combined extracts were dried over anhydrous magnesium sulfate, the drying agent was removed by filtration and chloroform evaporated under reduced pressure. The crude oily product was dissolved in acetone (4 mL) and left for crystallization in the refrigerator.

Di(biphenyl) (benzyloxycarbonylamino)(phenyl)methylphosphonate (**2m**); 33% yield; mp. 203-206^{0}C; ^{31}PNMR ($CDCl_3$): δ= 16.04 ppm.; ^{1}HNMR ($CDCl_3$): δ = 5.12 & 5.14 (2H, d, J_{AB} 12.0 Hz, C<u>H</u>$_2$Ph), 5.66 (1H, dd, J_{PH} 22.0 Hz, *J* 9.0 Hz, C<u>H</u>P), 6.07 (dd, 1H, *J* 9.0 Hz, *J* 4.0 Hz,N<u>H</u>), 6.09 – 7.91 (28H, m, Ar) ppm. HRMS (DMSO, TOF MS ESI$^+$): found 647.6399 [MNa$^+$], $C_{39}H_{32}NO_5PNa$ requires 648.6421.

Bis[4-(naphthyl-2-yl)phenyl] (benzyloxycarbonylamino)(phenyl)methylphosphonate (**2n**)

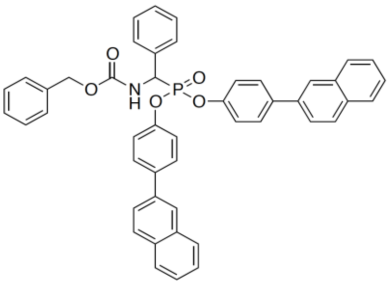

33% yield; mp. 196-199^{0}C; ^{31}PNMR ($CDCl_3$): δ= 15.89 ppm.; ^{1}HNMR (d_6DMSO): δ = 5.02 & 5.08 (2H, d, J_{AB} 12.0 Hz, C<u>H</u>$_2$Ph), 5.74 (1H, dd, J_{PH} 22.0 Hz, *J* 9.0 Hz, C<u>H</u>P), 7.21 – 7.92 (32H, m, Ar), 9.00 (1H, *J* 9.0 Hz, N<u>H</u>) ppm; ^{13}C NMR ($CDCl_3$): δ= 66.74, 53.47 (d, *J* 314.1 Hz), 117.28, 119.24, 119.28, 121.89, 121.95, 128.41, 128.78, 129.01, 129.13, 129.66, 129.71, 130.03, 130.38, 132.12, 132.31, 134.64, 136.96, 148.21,148.34, 148.44, 148.68 ppm.

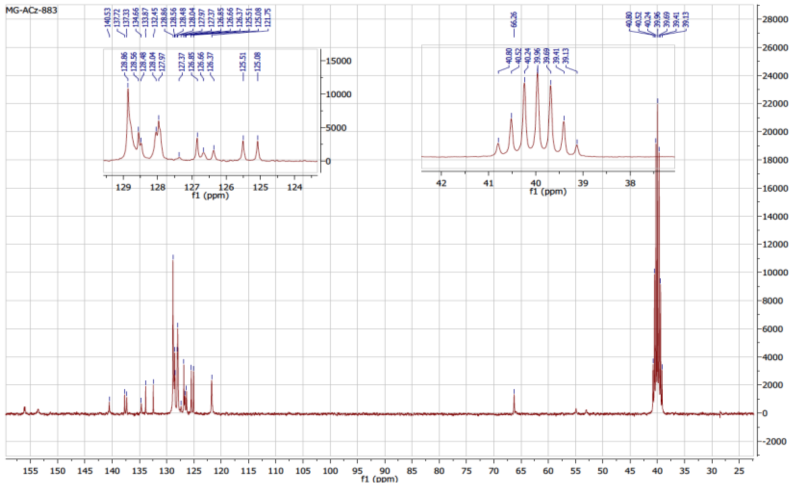

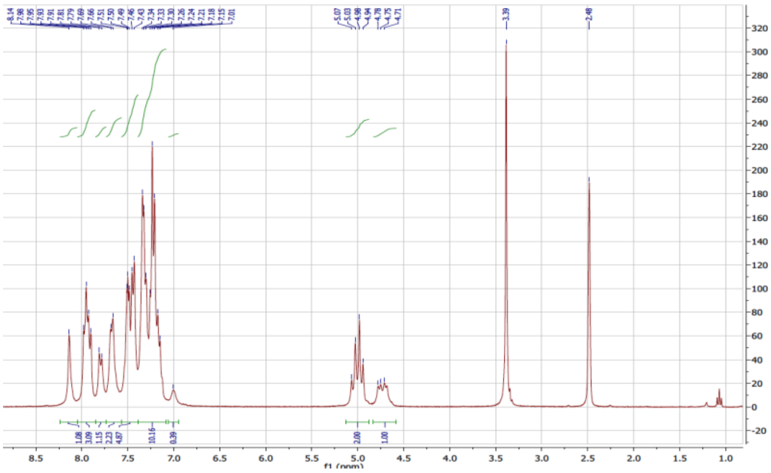

Bis(7-phenylnaphthalen-2-yl) (benzyloxycarbonylamino)(phenyl)methylphosphonate (**2o**)

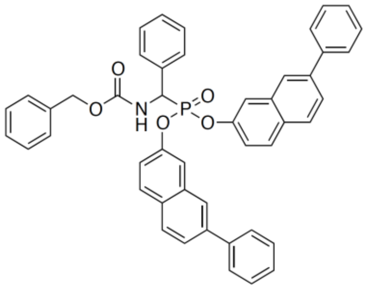

29% yield; mp. 238-240^{0}C decomp.; ^{31}PNMR ($CDCl_3$): δ= 16.13 ppm.; ^{1}HNMR (d_6DMSO): δ = 4.76 ppm (1H, bdd, J_{PH} 23.1 Hz, J 9.0 Hz, C<u>H</u>P), 4.96 & 5.05 (2H, d, J_{AB} 12.2 Hz, C<u>H</u>$_2$Ph), 7.01- 8.14 (33H, m Ar, N<u>H</u>) ppm; ^{13}C NMR ($CDCl_3$): δ= 66.26, 53.5 (d, J 285.4 Hz), 121.75, 125.08, 125.51, 126.37, 126.66, 126.86, 127.37, 127.97, 128.04, 128.48, 128.56, 128.86, 132.45, 133.87, 134.66, 135.84, 136.0, 137.33, 137.72, 140.53, 143.28, 156.00, 156.14 ppm. HRMS (DMSO, TOF MS ESI$^+$): found 726.2294 [MH$^+$], $C_{47}H_{37}NO_5P$ requires 726.7783.

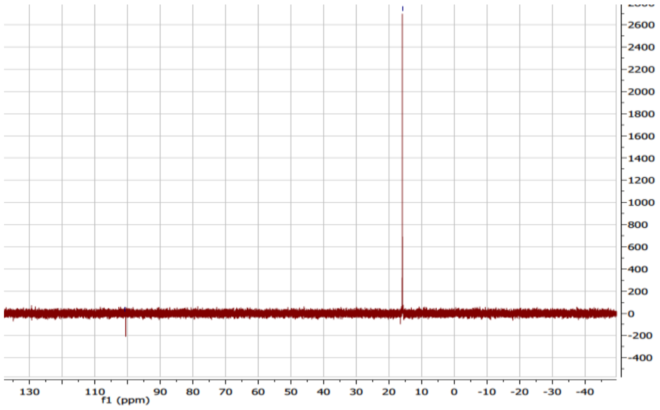

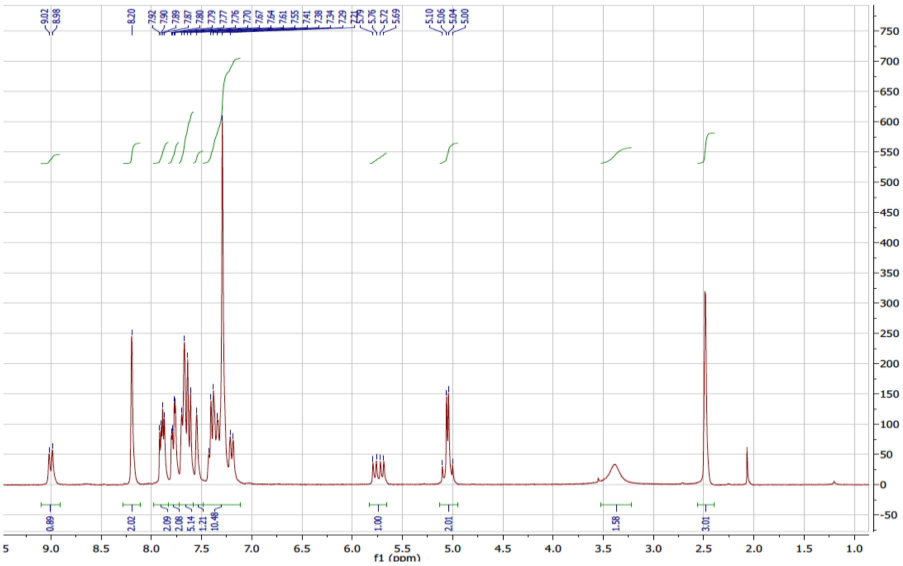

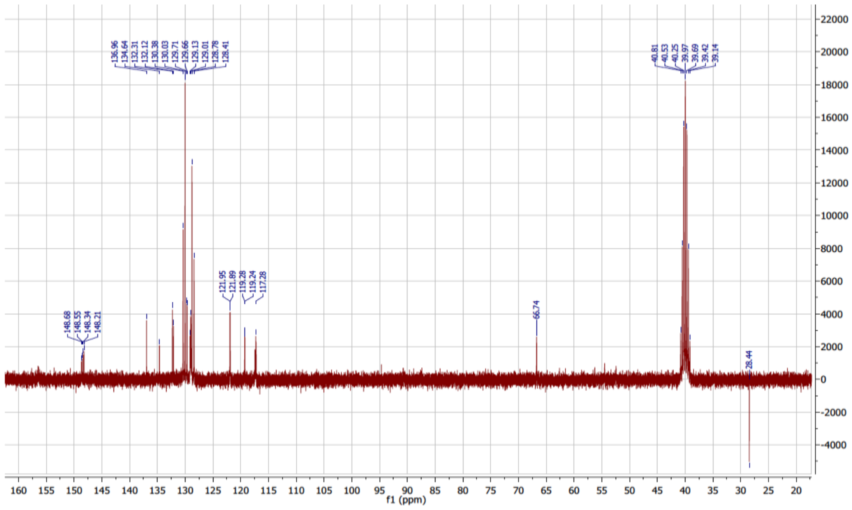

Bis(6-naphthylnaphthalene-2-yl) (benzyloxycarbonylamino)(phenyl)methylphosphonate (**2p**); 14.5% yield; mp. 244-246^{0}C decomp.; ^{31}PNMR (DMSO): δ= 15.78 ppm.; ^{1}HNMR

($CDCl_3$): δ = 5.08 & 5.14 (2H, d, J_{AB} 12.0 Hz, C<u>H</u>$_2$Ph), 5.74 (1H, bdd, J_{PH} 22.1 Hz, J 9.0 Hz, C<u>H</u>P), 5.96 (1H, bd, J 9.0 Hz, N<u>H</u>), 7.03-7.76 (36H, m, Ar) ppm.HRMS (DMSO, TOF MS ESI$^+$): found 825.6881 [MH^+], $C_{55}H_{40}NO_5P$ requires 826.8964.

Phenyl (benzyloxycarbonylamino)(phenyl)methylphosphonate (**4**)

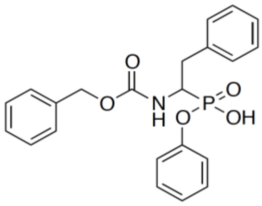

Diphenyl (benzyloxycarbonylamino)(phenyl)methylphosphonate (5.0 g, 10 mmol) and potassium hydroxide (5.8 g, 100 mmol) were suspended in a mixture of 1 M sodium hydroxide solution (20 mL) and dioxane (20 mL) and several crystals of 18-crown-6 were added (5–10 mg). The resulting mixture was refluxed for 10 min and left at room temperature for 24 h while stirring. Then the mixture was concentrated with a rotary evaporator, the aqueous residue was acidified to pH 1 with concentrated hydrochloric acid and extracted three times with 10 mL-portions of ethyl acetate. The organic fraction was dried over anhydrous sodium sulfate and the organic solvent was evaporated in vacuo. The resulting oil was dissolved in acetone (3 mL) and left for crystallization in the refrigerator (4 °C). In this manner 3.2 g of the desired product was obtained (81% yield); mp. 159-160^{0}C (lit.[25] m.p. 163-164^{0}C); ^{31}PNMR ($CDCl_3$): δ= 16.00 ppm; ^{1}HNMR ($CDCl_3$): δ = 5.00 & 5.10 (2H, d, J_{AB} 12.6 Hz, C<u>H</u>$_2$Ph), 5.14 (1H, dd, J_{PH} 22.3 Hz, J 10.1 Hz, C<u>H</u>P), 7.0-7.15 (15 H, m, Ar), 8.43 (dd, J 10.1 Hz & 2.8 Hz, N<u>H</u>); ^{13}C NMR ($CDCl_3$): δ= 53.6 (d, J 308.2 Hz, <u>C</u>HP), 66.27, 120.80, 120.85, 128.83, 128.85, 128.48, 128.51, 128.70, 128.78, 129.9, 136.84, 137.32, 151.33, 151.45, 156.38, 156.50. HRMS (DMSO, TOF MS ESI$^+$): found 397.1034 [MH^+], $C_{21}H_{20}NO_5P$ requires 397.3630.

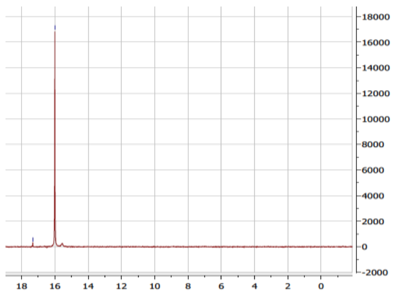

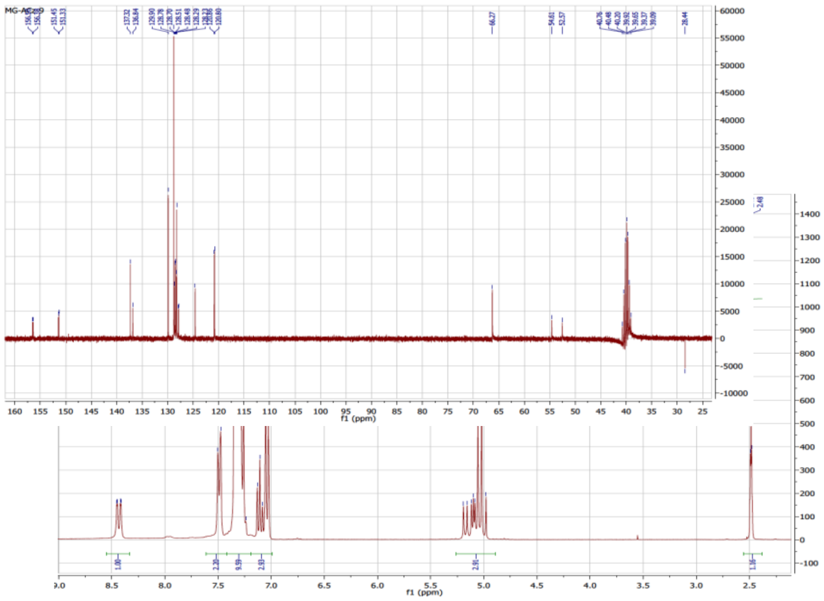

Phenyl (benzyloxycarbonylamino)(phenyl)methylphosphonic chloride (**5**)

Phenyl (benzyloxycarbonylamino)(phenyl)methylphosphonate (1 g, 2.5 mmol) was dissolved in dry chloroform (20 mL). This solution was warmed up to reflux and then thionyl chloride was added (3.65 ml, 5 mmol) and the mixture was refluxed for additional 2 h. Then volatile components of the reaction mixture were evaporated in vacuo, which resulted in 0.8 g (74%) of the desired product as dense oil. ^{31}PNMR ($CDCl_3$): δ= 33.32 & 33.79 (55:45) ppm; ^{1}HNMR ($CDCl_3$): δ = 5.02 & 5.14 (2H, d, J_{AB} 10.0 Hz, C<u>H</u>$_2$Ph), 5.34 (1H, dd, J_{PH} 22.0 Hz, J 9.0 Hz, C<u>H</u>P), 5.81 (1H, bd, J 9.0 Hz, N<u>H</u>), 6.99-7.78 (15H, m, Ar).

***Phenyl (benzyloxycarbonylamino)(phenyl)methylphosphonic bromide* (6)**

This compound was obtained by using the procedure identical as described above for the synthesis of compound **5**. 0.5 g (42% yield) of the desired bromide as a yellowish oil was obtained. ^{31}PNMR ($CDCl_3$): δ= 32.09 & 32.64 (58:42) ppm; ^{1}HNMR ($CDCl_3$): δ = 4.88 & 4.97 (2H, d, J_{AB} 10.0 Hz, C$\underline{H}_2$Ph), 5.07 (1H, dd, J_{PH} 23.8 Hz, J 9.0 Hz, C$\underline{H}$P), 5.74 (1H, bd, J 9.0 Hz, N$\underline{H}$), 6.71-7.38 (15H, m, Ar).

Mixed aliphatic-phenyl esters of (benzyloxycarbonylamino)(phenyl)methylphosphonic acid (**7a–f**)

Phosphonic chloride 5 (0.5 g, 1 mmol) was dissolved in alcohol (10 mmol) and refluxed for 3 h. Then the solvents were evaporated under reduces pressure and the resulting oil was dissolved in acetone (3 ml) and left at 4^{0}C for crystallization or purified by silica gel column chromatography using mixture of hexane-ethyl acetate (4:6 v/v) as eluent.

n-Pentyl phenyl (benzyloxycarbonylamino)(phenyl)methylphosphonate (**7a**)

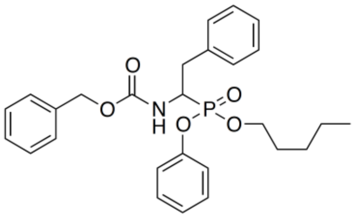

79% yield; mp. 124-127^{0}C; ^{31}PNMR ($CDCl_3$): δ= 18.31 & 18.36 (23:77 after crystallization) ppm.; ^{1}HNMR ($CDCl_3$): δ = 0.76 (3H, t, J 7.0 Hz, C$\underline{H}_3$), 1.11-1.21 (4H, m, 2xC$\underline{H}_2$), 1.30-1.50 (2H, m, C$\underline{H}_2$), 3.80-4.12 (2H, m, OC$\underline{H}_2$), 4.91 & 5.10 (major) and 5.06 & 5.11 (minor) (2H, d, J_{AB} 12.3 Hz, C$\underline{H}_2$Ph), 5.30 (dd, J_{PH} 22.1 Hz, J 9.2 Hz, C$\underline{H}$P), 6.90-7.50 (15H, m, Ar); 8.69 (d, J 9.2 Hz, N$\underline{H}$); ^{13}C NMR ($CDCl_3$): δ= 14.26, 22.01, 27.35, 28.48, 30.00 (d, J 8.5 Hz), 53.03 (d, J *311.*1 Hz, $\underline{C}$HP), 67.47 (d, J 12.2 Hz), 120.73, 120.78, 128.34, 128.41,

128.74, 128.83, 130.14, 135.52, 137.20, 150.79, 150.92, 156.41, 156.52; HRMS (DMSO, TOF MS ESI$^+$): found 490.1768 [MNa^+], $C_{26}H_{30}NO_5PNa$ requires 490.4861.

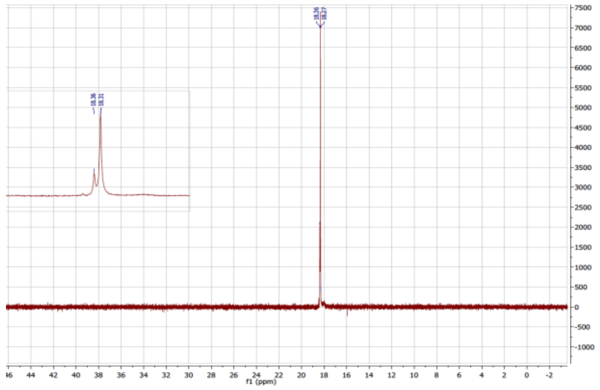

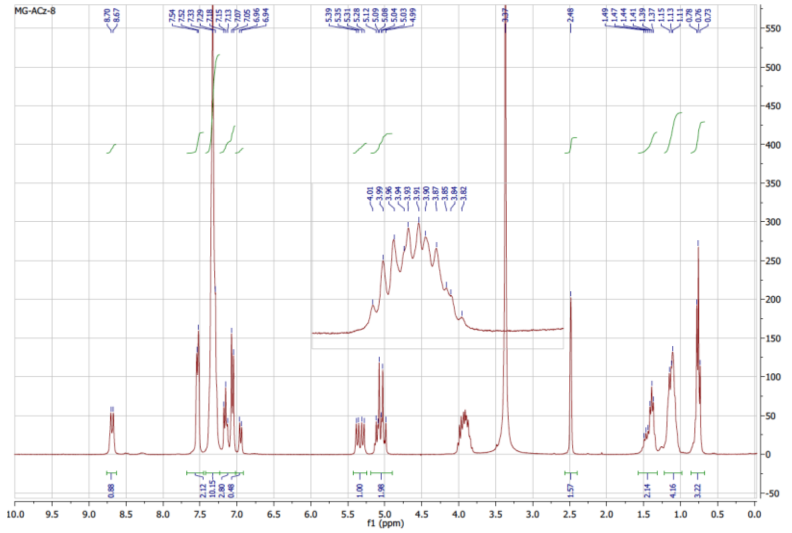

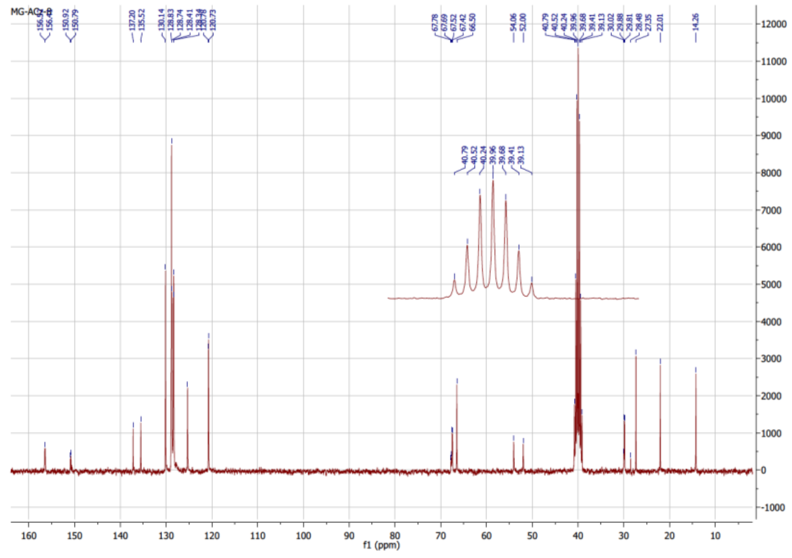

n-Undecyl phenyl (benzyloxycarbonylamino)(phenyl)methylphosphonate (**7b**); 54.5% yield; mp. 148-150^{0}C; ^{31}PNMR ($CDCl_3$): δ= 17.81 & 18.04 (15:85 by chromatography) ppm; ^{1}HNMR ($CDCl_3$): δ = 0.89 (3H, t, *J* 7.0 Hz, C<u>H</u>$_3$), 1.12-1.65 (18H, m, 9xC<u>H</u>$_2$), 1.30-1.50 (2H, m, C<u>H</u>$_2$), 3.61-3.94 (2H, m, OC<u>H</u>$_2$), 5.07 & 5.17 (2H, d, J_{AB} 12.0 Hz, C<u>H</u>$_2$Ph), 5.34 (dd, J_{PH} 22.0 Hz, *J* 8.9 Hz, C<u>H</u>P), 5.91 (d, *J* 8.9 Hz, N<u>H</u>), 7.12-7.46 (15H, m, Ar).

n-Docosyl phenyl (benzyloxycarbonylamino)(phenyl)methylphosphonate (**7c**); 27% yield; mp. 167-169^{0}C; ^{31}PNMR ($CDCl_3$): δ= 18.09 & 18.65 (10:90 by chromatography) ppm; ^{1}HNMR ($CDCl_3$): δ = 0.79 (3H, t, *J* 8.0 Hz, C<u>H</u>$_3$), 1.26-1.75 (40H, m, 20xC<u>H</u>$_2$), 3.71-4.03 (2H, m, OC<u>H</u>$_2$), 5.24 & 5.34 (2H, d, J_{AB} 12.0 Hz, C<u>H</u>$_2$Ph), 5.56 (dd, J_{PH} 22.0 Hz, *J* 9.0 Hz, C<u>H</u>P), 5.79 (d, *J* 9.0 Hz, N<u>H</u>), 7.33-7.89 (15H, m, Ar).

3-Methoxyethyl phenyl (benzyloxycarbonylamino)(phenyl)methylphosphonate (**7d**)

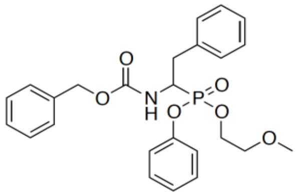

72.5% yield; mp. 110-113^{0}C ; ^{31}PNMR ($CDCl_3$): δ = 19.12 & 19.18 (65:35 by chromatography) ppm; ^{1}HNMR ($CDCl_3$): δ = 3.17(major) and 3.20 (minor) (3H, s, C<u>H</u>$_3$), 3.37-3.42 (major) and 3.35-3.45 (minor) (2H, m, C<u>H</u>$_2$OCH$_3$), 4.00-4.15 (2H, m, OC<u>H</u>$_2$), 5.02 & 5.10 (major) and 5.06 & 5.12(minor) (2H, d, J_{AB} 12.0 Hz, C<u>H</u>$_2$Ph), 5.38 (1H, dd, J_{PH} 21.0 Hz, *J* 9.0 Hz, C<u>H</u>P), 6.95-7.55 (15H, m, Ar), 8.66 (major) and 8.70 (minor) (1H, d, *J* 9.0 Hz, N<u>H</u>); ^{13}C NMR ($CDCl_3$): δ= 53.04 (minor) and 53.05 (d, *J* 314.1 Hz, <u>C</u>HP), 58.45, 66.59 (major) and 66.61 (minor) (d, *J* 12.0 Hz), 66.85 (d, *J* 12.2 Hz), 71.23 (d, *J* 8.5 Hz), 120.69, 120.75, 125.32, 128.39, 128.77, 128.85, 130.14, 135.54, 135.65, 137.22, 137.23,

150.69, 150.82, 156.36, 156.51; HRMS (DMSO, TOF MS ESI^{+}): found 478.1396 [MNa^{+}], $C_{24}H_{26}NO_6PNa$ requires 478.4322.

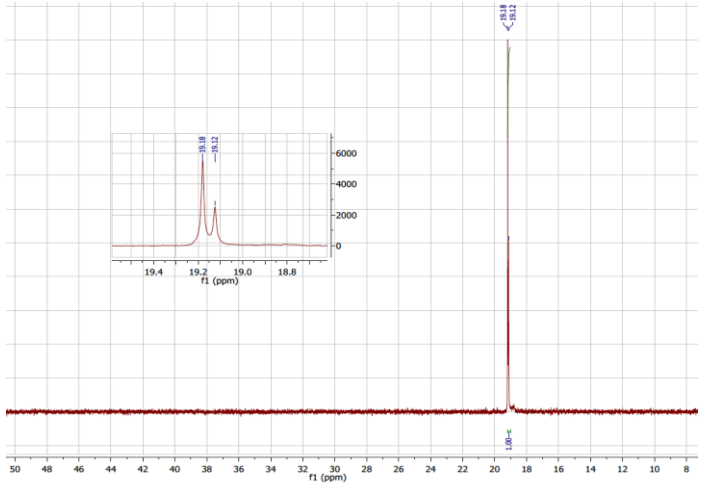

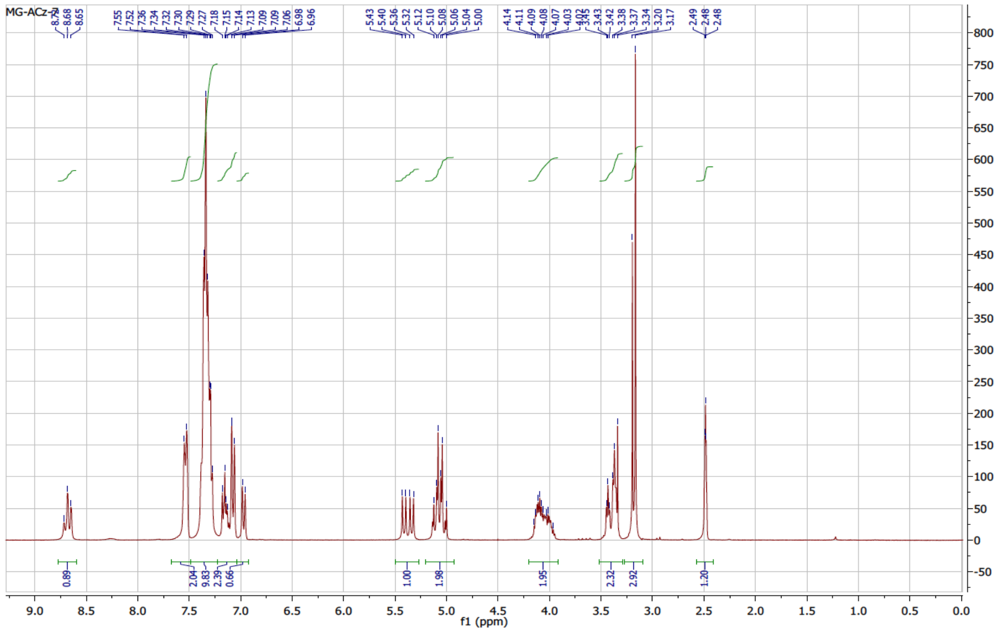

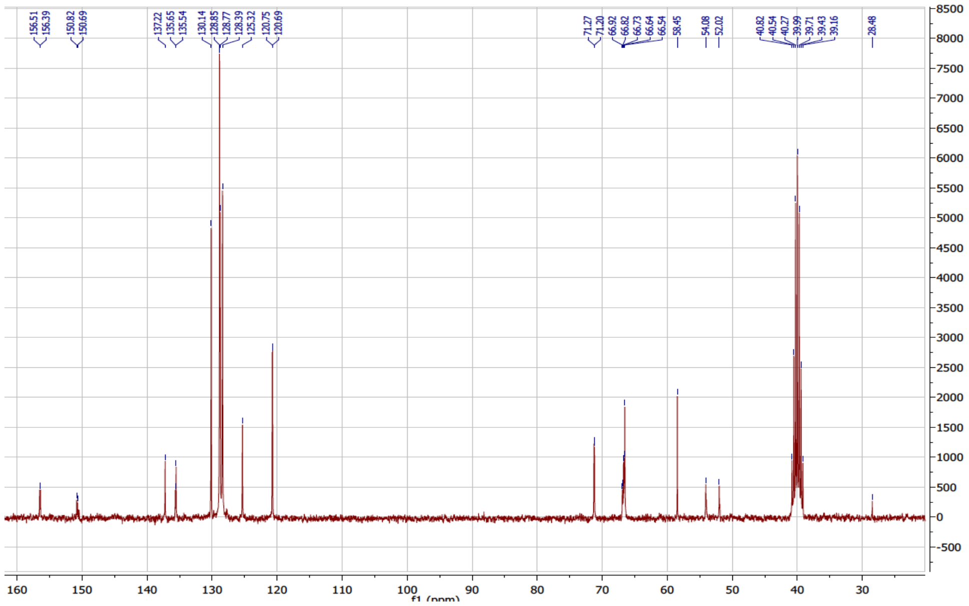

3,6,9-Trioxaundecyl phenyl (benzyloxycarbonylamino)(phenyl)methylphosphonate (**7e**); 57.5% yield; mp. 71-78^{0}C ; ^{31}PNMR ($CDCl_3$): δ= 19.07 & 19.13 (41:59 by chromatography) ppm; ^{1}HNMR ($CDCl_3$): δ = 1.05 (3H, t, *J* 7.1 Hz, CH_3), 3.30-3.55 (24H, m, 12xCH_2), 3.90-4.10 (2H, m, CH_2O), 5.06 (major) & 5.07 (minor) (2H, AB system, J_{AB} Hz, 12.0Hz), 5.38 (1H, dd, J_{PH} 24.0 Hz, *J* 9.0 Hz, C<u>H</u>P), 7.00-7.60 (15H, m, Ar), 8.65 (1H, t, *J* 9.0 Hz, N<u>H</u>); ^{13}C NMR ($CDCl_3$): δ= 15.58, 53.3 (d, *J* 315.6 Hz, <u>C</u>HP), 66.05, 66.51, 69.70, 70.11, 120.72, 120.77, 125.33, 128.37, 128.77, 238.85, 130.13, 135.51,

135.65, 137.20, 150.43; HRMS (DMSO, TOF MS ESI$^+$): MH$^+$, found 536.1814 [MNa$^+$], $C_{27}H_{32}NO_7PNa$ requires 536.5116.

4,8-Dioxaduodecyl phenyl (benzyloxycarbonylamino)(phenyl)methylphosphonate (**7f**); 51% yield; mp. 102-104^{0}C ; ^{31}PNMR ($CDCl_3$): δ= 17.72 & 17.79 (40:60 by chromatography) ppm; ^{1}HNMR ($CDCl_3$): δ = 0.79 (3H, bt, *J* 6.8 Hz, CH_3), 1.12-1.22 (4 H, m, 2xCH_2), 1.43-1.67 (12H, m, 6xCH_2), 3.68-5.02 (26H, m, 13xCH_2), 5.09 (major) & 5.17 (minor) (2H, AB system, J_{AB} Hz, 12.0Hz), 5.42 (major) & 5.42 (minor) (1H, dd, J_{PH} 24.0 Hz, *J* 9.0 Hz, C<u>H</u>P), 6.03 (1H, m, N<u>H</u>), 7.24-7.89 (15H, m, Ar).

Mixed aromatic-phenyl esters of (benzyloxycarbonylamino)(phenyl)methylphosphonic acid (**7g,h**)

Phosphonic chloride 5 (1.07 g, 2.5 mmol) was dissolved in dry chloroform (20 ml) and corresponding alcohol (5 mmol) was added. This solvent was heated up to boiling and then triethylamine (0.18 ml, 2.6 mmol) was added dropwise. The resulting solution was additionally refluxed for 5 h. Then the solvents were evaporated under reduces pressure and the resulting brown oil was dissolved in chloroform and left for crystallization. Second portion of product was purified by flash chromatography using a gradient of hexane and chloroform (solvent containing 5% of chloroform more after each 10 minutes of elution) as eluent.

2-Naphthyl phenyl N-(benzyloxycarbonylamino)(phenyl)methylphosphonate (**7g**); 33% yield; mp. 156-159^{0}C ; ^{31}PNMR ($CDCl_3$): δ= 14.73 ppm; ^{1}HNMR ($CDCl_3$): 5.16 (2H, AB system, J_{AB} Hz, 12.1 Hz), 5.68 (1H, dd, J_{PH} 21.8 Hz, *J* 10.6 Hz, C<u>H</u>P), 5.98 (1H, bd, *J* 10.6 Hz, N<u>H</u>), 6.88-7.69(22H, m, Ar).

Anthracen-9-ylmethyl phenyl (benzyloxycarbonylamino)(phenyl)methylphosphonate (**7h**); 4% yield; mp. 124-126^{0}C; ^{31}PNMR ($CDCl_3$): δ= 13.50 ppm; ^{1}HNMR ($CDCl_3$): 5.01 (2H, AB system, J_{AB} Hz, 12.2 Hz), 6.31 (1H, bdd, C<u>H</u>P), 7.00-7.41(24H, m, Ar), 8.12 (1H, bs, N<u>H</u>).

Fluorescence studies

Solutions of the studied compounds in DMSO (0.05–0.1 mmole) were prepared in Eppendorf tubes (300 µL). These solutions were transferred to wells of CELLSTAR geiner black 96 wells plate and were irradiated with 254 and 366 nm UV light. The fluorescence was observed visually and presented schematically in Figure S1. Photographic documentation (Olympus MJU) of the results is not fully reasonable and therefore also negative pictures have been analyzed with moderate success (Figure S1).

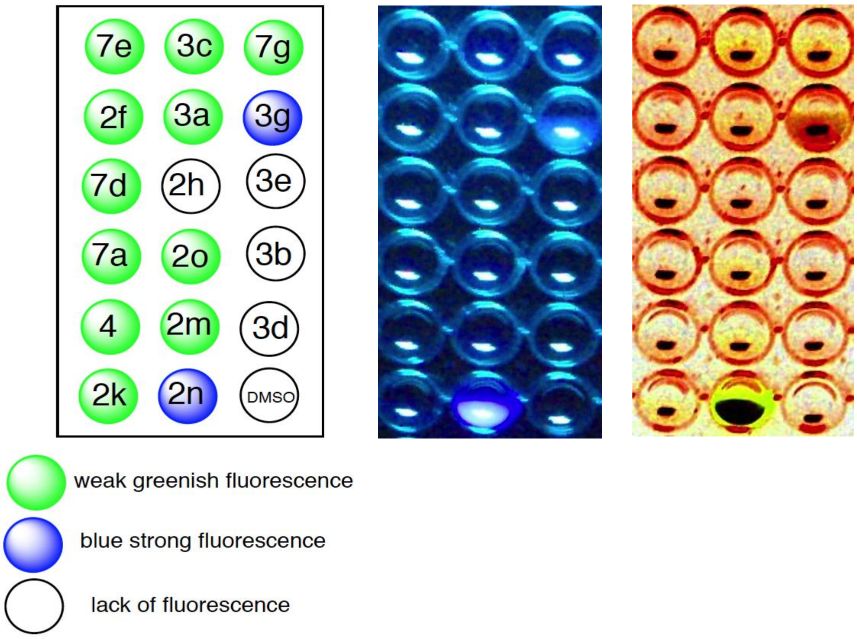

Figure S1. Examination of fluorescent properties of synthesized compounds

References

1. Russel, M. A. Ph.D. Dissertation, University of Huddersfield, 2007.

2. Wenska, M.; Jankowska, J.; Sobkowski, M.; Stawiński, J.; Kraszewski, A. T*etrahedron Lett.* **2001**, *42*, 8055-8058.

3. Sieńczyk, M.; Winiarski, Ł.; Kasperkiewicz, P.; Psurski, M.; Wietrzyk, J.; Oleksyszyn, J. *Bioorg. Med. Chem. Lett.* **2011**, *21*, 1310-1314.

4. Oleksyszyn, J.; Subotkowska, L.; Mastalerz, P. *Synthesis* **1979**, 985-986.
